# Practical experience commissioning MRI‐compatible tandem and ring applicators for use with the Bravos HDR afterloader

**DOI:** 10.1002/acm2.14094

**Published:** 2023-07-19

**Authors:** Jessica M. Fagerstrom

**Affiliations:** ^1^ Radiation Oncology University of Washington Seattle Washington USA; ^2^ Kaiser Permanente Seattle Washington USA

**Keywords:** brachytherapy, commissioning, HDR, image guidance, MRI, quality assurance, tandem and ring

## Abstract

Five complete MR‐conditionally approved ring sets, including fifteen tandems, and two additional rings, were commissioned at an institution intending to use them in an MRI planning environment with a Bravos HDR brachytherapy remote afterloader. Channel length, radiograph, autoradiograph, ring offset, and treatment interrupt measurements were performed, and applicators were assessed in both CT and MRI. During commissioning, one ring was found to be defective and was returned to the manufacturer for a replacement. The eventual complete applicator suite (including the replacement ring) was found to follow the manufacturer‐provided specifications, including those delineated in vendor‐provided 3D virtual models and those defined within the manufacturer's instructions for use documentation. Based on this work, an offset correction of −0.4 cm will be used for all tested rings using the Bravos system's internal distal dwell position correction feature during treatment preparation. This study reiterated the requirement for careful commissioning of each applicator intended for clinical service considering the intended use and the planned clinical environment and work processes.

## 1 INTRODUCTION

The Varian Bravos high‐dose‐rate (HDR) brachytherapy remote afterloading system (Varian Medical Systems, Inc., Palo Alto, CA) was released commercially in 2018. The platform's major characteristics have been previously characterized in the literature.^1^ A suite of MRI‐compatible tandem and ring applicators was purchased for use with the Bravos system, with the intent to use MRI data for treatment planning acquired with the implant in place (denoted here as “MRI‐based” planning^2^). Previous work has shown the importance of acquiring accurate commissioning measurements to characterize each HDR applicator in clinical service.^3^, ^4^, ^5^, ^6^ Prior to clinical use, testing was completed to define basic physical characteristics of the applicators.

AAPM's TG‐303 report contains guidance on the use of MRI in HDR brachytherapy,^7^ and considers MRI to be the gold standard for target volume delineation in gynecologic HDR applications, while high‐resolution CT to be the gold standard for geometric accuracy. Accurate three‐dimensional applicator reconstruction is essential for high‐quality treatment planning, and the measurements described here support confidence in reconstruction and subsequent dose calculations. Additionally, offset characterization is described, and a discussion is included regarding how these offset measurements may be used in clinical practice.

## 2 METHODS

**FIGURE 1:**
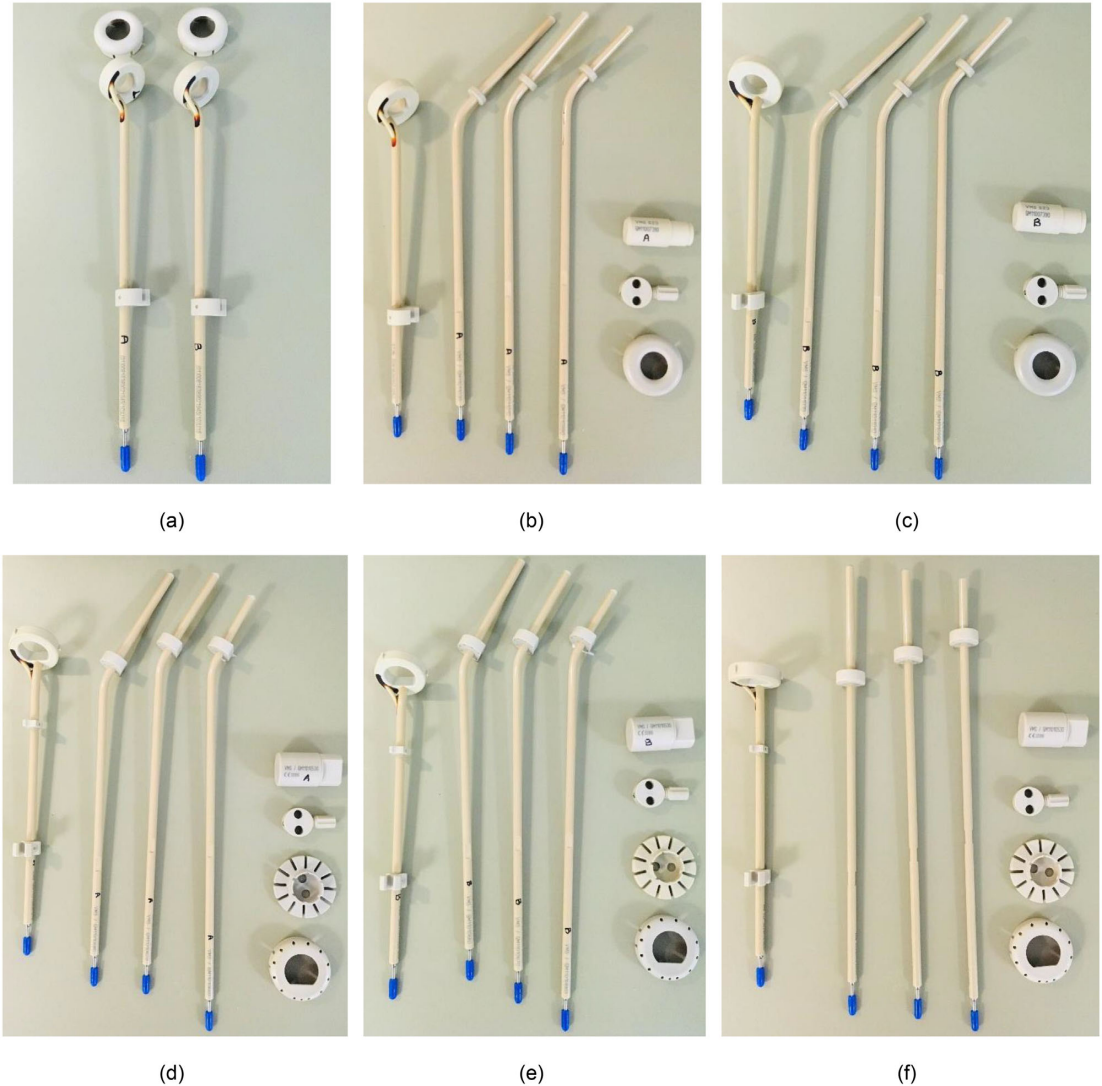
Photographs of the commissioned tandem and ring components. Components include (a): Set A and Set B of 45°, ⌀26 mm × 32 mm rings and corresponding Set A and Set B 36 mm caps; (b): complete Set A 45°, ⌀30 mm × 36 mm; (c): complete Set B 45°, ⌀30 mm × 36 mm; (d): complete Set A 3D 60°, ⌀30 mm × 36 mm; (e): complete Set B 3D 60°, ⌀30 mm × 36 mm; and (f): complete 3D 90°, ⌀30 mm × 36 mm.

Five complete Varian MRI‐compatible ring applicator sets were purchased for use with a Varian Bravos HDR afterloader. The applicators are composed of polyetheretherketone (PEEK) and titanium, and are deemed by the manufacturer to have MR conditional status. Two complete 45° ring sets (part number GM11010490), two complete 3D interstitial 60° ring sets (GM11010190), and one complete interstitial 90° ring set (GM11010290) were purchased. Each set included the ⌀30 mm × 36 mm ring probe, three intrauterine tandems of different lengths, a centering joint with clamping screw, distance cap, and a disassembly pin. The three interstitial sets included needle collectors. In addition to the complete sets, two additional ⌀26 mm × 32 mm ring probes were also purchased with distance caps. Internally, duplicate components were denoted “Set A” and “Set B.” Assessments regarding channel length, radiographs, autoradiographs, ring offset distances, treatment interrupts, CT data with vendor‐provided solid models, and MRI data were completed prior to releasing the applicators for clinical use. Images of the commissioned applicators are included in Figure 1.

### 2.1 Channel length assessment

The length of each applicator channel was assessed using both Varian's Length Assessment Device (LAD) as well as the afterloader's internal length measurement system. Both measurement tools must be used with transfer guide tubes. The LAD has demarcations at 0.5 cm intervals, and applicator channel length estimates were acquired to the nearest 1 mm. On the afterloader, plans were created to send the source to a distal position of 131.9 cm (the furthest possible distal dwell position for a nominal 132 cm total length applicator and transfer guide tube combination). The dummy and source were sent to the most distal position on each applicator. For the Bravos platform, the system will report a total required shift for each applicator and transfer guide tube combination. The mean correction magnitude for the distal‐most dwell position was recorded for each combination of transfer guide tube and applicator, with no push test completed during these tests.

### 2.2 Radiographs and autoradiographs

**FIGURE 2:**
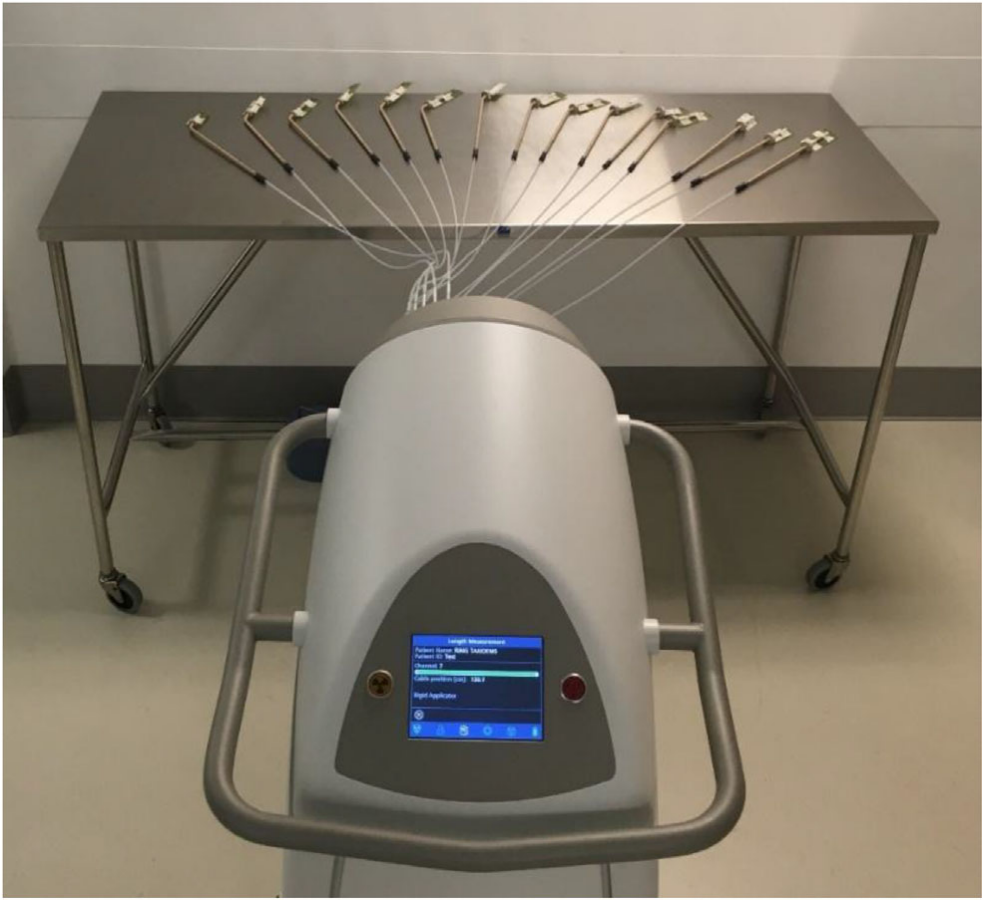
Tandem applicators with EBT3 film in place are shown in position for autoradiograph acquisition.

Radiographic images were taken of the applicators using imaging systems integrated on a Varian TrueBeam linear accelerator. The kV on‐board imaging system (50 kV, 40 mA, 40 ms, small focal spot size) was used to acquire images with the applicators nominally at 50 cm from isocenter and in contact with the imager to assess for voids or other defects. Radiographs were also acquired of the tandem applicators using EBT3 radiochromic film (Ashland Inc., Bridgewater, NJ), placed at 100 cm SDD/SSD. The film was fixed to the tandem applicators immediately above the position of the cervical stop. A 12 MeV electron beam was used to deliver 600 MU to the film with the applicators taped in place in order to mark the outer dimensions of the applicators on the film. An uncoded marker wire was placed in each tandem for these irradiations. The film was then irradiated on the Bravos system for autoradiographic documentation, programming source positions at 1 cm intervals at a nominal 8‐s dwell time for each position (assuming a 10 Ci source). The Bravos tandem film irradiation geometry is shown in Figure 2.

### 2.3 Offset distance quantification

Dwell position accuracy, including within the ring applicator, is necessary for robust treatment planning. It has been confirmed in the literature that the trajectory of the source wire along the lumen in ring applicators can undergo noncircular “snaking,” and it is recommended that each ring applicator be individually characterized for an offset correction.^8^, ^9^, ^10^ The required positional offset was measured and verified during this commissioning process. Preliminary measurements were completed following Varian's recommendations included in their instructions for use documentation,^11^ and then initial values from this process were used to verify the final offset.

Radiochromic EBT3 film was used to acquire radiographs and autoradiographs on the rings following the same process as used for the tandems, as described in the previous section. Film was attached to the applicators parallel to the ring plane, and rings were placed directly on the kV imaging panel of a Varian TrueBeam linear accelerator, positioned at 100 cm SDD/SSD, with uncoded marker wires in place. A 12 MeV electron beam was used to deliver 800 MU to the film in order to mark the outer dimensions of the applicators on the film. Without moving the film, autoradiographs were then acquired using the BRAVOS afterloader with programmed source positions at the distal‐most allowable position (131.9 cm), with a short dwell time (0.4 s nominal time for a 10 Ci source), and then subsequent dwells located in 1 cm increments (each nominally 8 s) to encompass the entire ring. Note that the PEEK/titanium ring applicators used in this study feature a closed circular design. The outer dimension of the ring distal end cannot be marked directly on the film, as with similar titanium open rings. The outer dimension of the distal‐most end of the lumen must be determined via the radiograph. The marking of the distal‐most dwell position, as noted on the radiograph, was reinforced using a thin‐tipped permanent marker. The autoradiographs were then scanned using an EPSON 10000XL white‐light flatbed document scanner (Epson America, Long Beach, CA) and imported into Eclipse Treatment Planning System (Varian). The angle between the tip of the lumen and the center of the radiation source footprint, with respect to the center of the ring, was measured and used to calculate the path length and average offset correction for each ring, using the equation from Varian's instructions for use documentation:

$$\mathrm{Path}\;\mathrm{length}\;=\;\left(\pi \;r\;\frac{\alpha }{180^{\circ }}\right)-\mathrm{SCDT}$$

where *r* is the radius of the ring, *ɑ* is the angle between the tip of the lumen and the center of the radiation source footprint, and *SCDT* is the source‐center distance to the tip of the source cable. For Bravos, *SCDT* is 0.25 cm. The equation was used to follow Varian's procedure to find the shift distance by comparing the planned position to the delivered position. This shift distance was verified using repeated radiograph/autoradiograph measurements following the previously described procedure.

### 2.4 Ring interrupt testing

The Bravos system sends the source wire out to the distal‐most programmed position, then successively pulls the wire back for the next‐most distal position, repeating this process until all dwells have been treated. If treatment is interrupted, the source wire will only be sent out to the remaining distal‐most dwell position to restart treatment. There was a concern that this process could negatively impact positional accuracy in interrupted ring treatments due to snaking within the circular lumen in the rings. To verify that treatment interrupts would not negatively impact positioning accuracy, interrupt testing was completed on all rings using the same radiograph/autoradiograph process as previously described including the 4 mm distal offset, but with a treatment interrupt initiated at the worst possible scenario: immediately after the first significant (8‐s) dwell began at the 131.4 cm position (immediately following the completion of the 0.4‐s dwell at the 131.9 cm position).

### 2.5 CT data and treatment planning system

Applicators were imaged using a GE LightSpeed RT16 scanner (General Electric Healthcare, Milwaukee, WI) with a technique of 120 kV, 97 mA, 0.625 mm thickness, and a field of view of 25.0 cm (the CT protocol currently used for gynecologic HDR patient imaging). Applicators were positioned with long axes approximately parallel to the CT imaging axis, fixing the applicators such that the curved portion of the applicators was suspended in air using a container of dry rice. The CT data was imported into the treatment planning system for review. The tandem and ring applicators are intended to be used clinically with the vendor‐provided digital solid models in the treatment planning system, BrachyVision (Varian) v.15.5. The solid model files were downloaded from the vendor's document library on myVarian.com and imported into BrachyVision. The solid models overlaid with the applicator CT data were reviewed by an experienced certified medical dosimetrist and medical physicist.

### 2.6 MRI data

**FIGURE 3:**
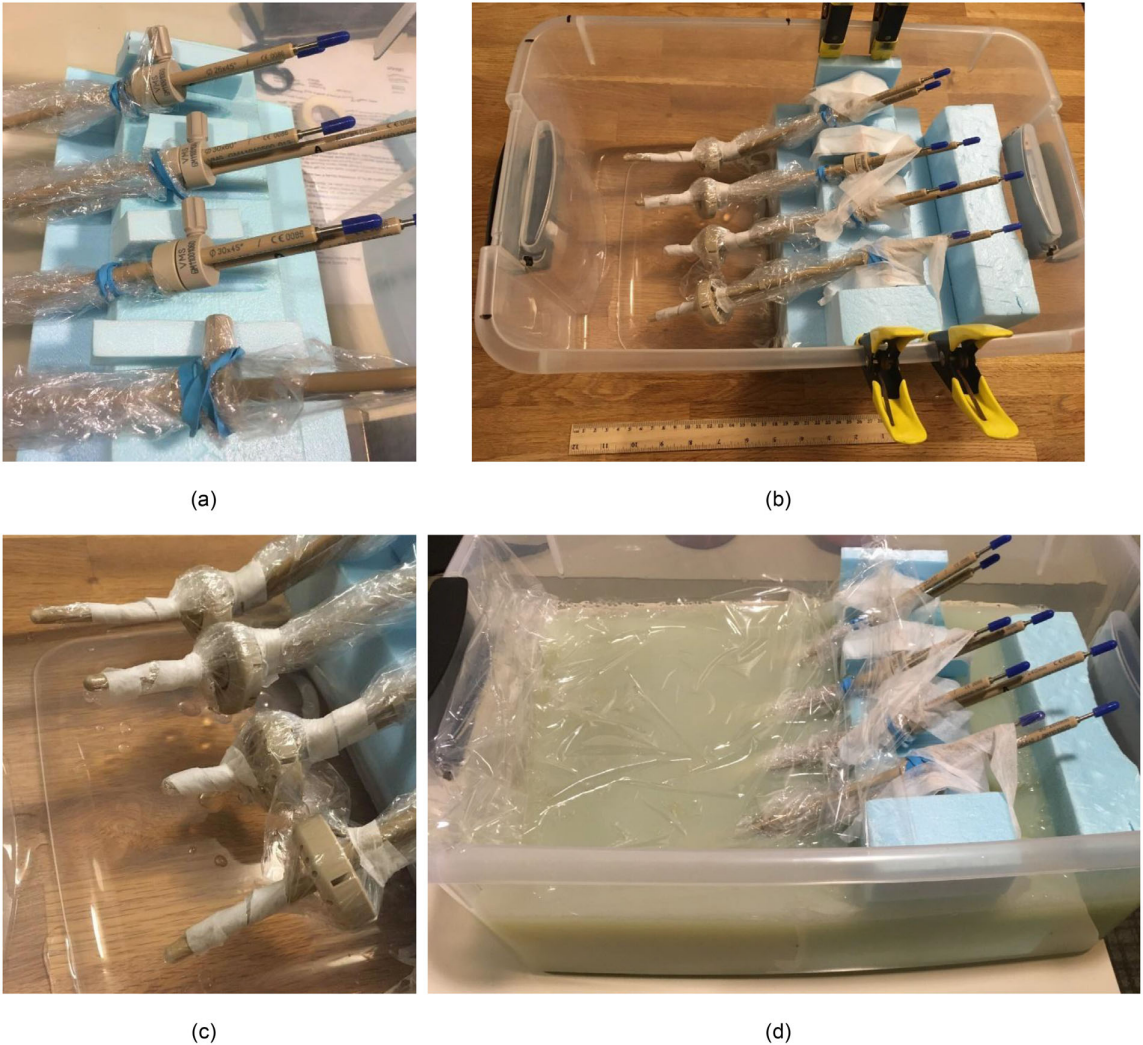
Photograph of the phantom for MR imaging of the rings. A close‐up view of the prepared applicator handles including protective blue cleaning caps is shown in (a), the phantom prepared for gel is shown in (b), a close‐up view of the applicator tips is shown in (c), and the completed phantom is shown in (d). From top to bottom, the four applicator groupings are: (1) Set A 45°, ⌀26 mm × 32 mm ring with its associated ring cap and the 45°× 80 mm tandem; (2) Set A 45°, ⌀30 mm × 36 mm ring with its associated ring cap and the 45°× 60 mm tandem; (3) Set A 60°, ⌀30 mm × 36 mm ring with its associated ring cap and the 60°× 40 mm tandem; and (4) 90° ⌀30 mm × 36 mm ring with its associated ring cap and the 90°× 60 mm tandem.

All the components included in this report are noted as conditionally approved for MRI by the vendor, and they received approval from the institution's MR safety team prior to the start of commissioning measurements. MRI artifacts and distortion may be influenced by several variables, including array coils, pulse sequences, gradient distortion corrections, and field strength, so MR imaging is a recommended commissioning step prior to applicator use in patients. Phantoms using rice to place applicators in a suspended position will not generate a signal in MRI, and water is prone to vibrational effects and exhibits a long settling time, so a custom gel phantom was constructed (Figure 3). Four rings and four tandems were imaged suspended in phantom, in a positional geometry designed to be similar to future patient treatments. The gel was created using ratios of 1 L distilled water, 30 g agar‐agar powder, and 5 mL of 0.1 CuSO_4_ solution following the process described by Fagerstrom and Kaur,^12^ based on work by Haack et al.^13^ and Mitchell et al.^14^ The applicators were protected using a thin layer of plastic wrap before pouring the agar solution into the phantom base. This plastic introduced an air gap immediately surrounding the applicators, though this volume was minimized to the extent possible. The phantom was scanned using a 1.5 T MAGNETOM Aera scanner (Siemens Healthcare, Erlangen, Germany) with body flex receiver coils, with the MRI protocol developed for gynecologic HDR patients. The center of the phantom was positioned approximately at MRI scanner isocenter, and sequences included 3D, T2‐weighted 1‐mm isotropic pixel size scans at 440 Hz sequence readout.

## 3 RESULTS

### 3.1 Channel length assessment

The channel length for each applicator was assessed using both the LAD and the internal afterloader length measurement. Repeated measurements with the LAD indicated each tandem and transfer guide tube combination had a length of 132.0 cm ± 0.0 cm, and each ring and transfer guide tube combination had a length of 132.1 cm ± 0.1 cm, with the reported uncertainty the Type A uncertainty associated with repeated measurements performed with each applicator and available transfer guide tube combination. It is noted that the Length Assessment Device has some amount of play within the ring applicators. For the afterloader length measurements, for all deployments, the afterloader reported between 0.0 and 0.2 cm absolute shift from the planned value of 131.9 cm for all transfer guide tube/applicator combinations. The mean correction magnitude for the most distal dwell position reported by the afterloader, averaged over all deployments, was 0.05 cm ± 0.06 cm, or 0.1 cm ± 0.1 cm reported to the correct number of significant figures.

### 3.2 Radiographs and autoradiographs

**FIGURE 4:**
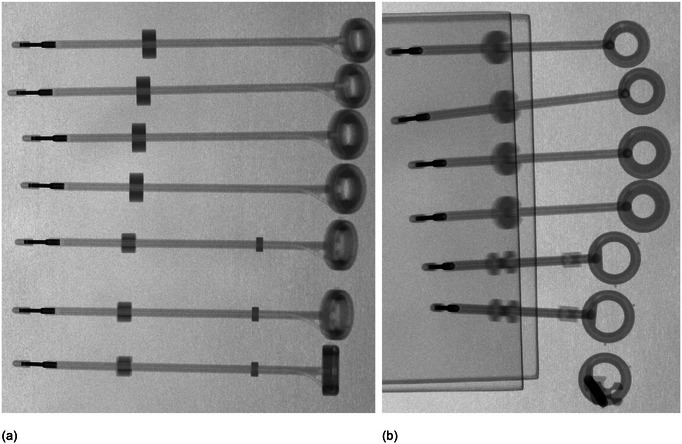
Radiographs of the ring applicators, with no marker wires in place. Applicators are arranged from top to bottom, Set A 45° ring ⌀26 mm × 32 mm, Set B 45° ring ⌀26 mm × 32 mm, Set A 45° ring ⌀30 mm × 36 mm, Set B 45° ring ⌀30 mm × 36 mm, Set A 3D 60° ring ⌀30 mm × 36 mm, Set B 3D 60° ring ⌀30 mm × 36 mm, and 3D 90° ring ⌀30 mm × 36 mm. The rings are arranged lying directly on the imager in (a), and propped such that the ring surface is flush with the imager surface in (b).

**FIGURE 5:**
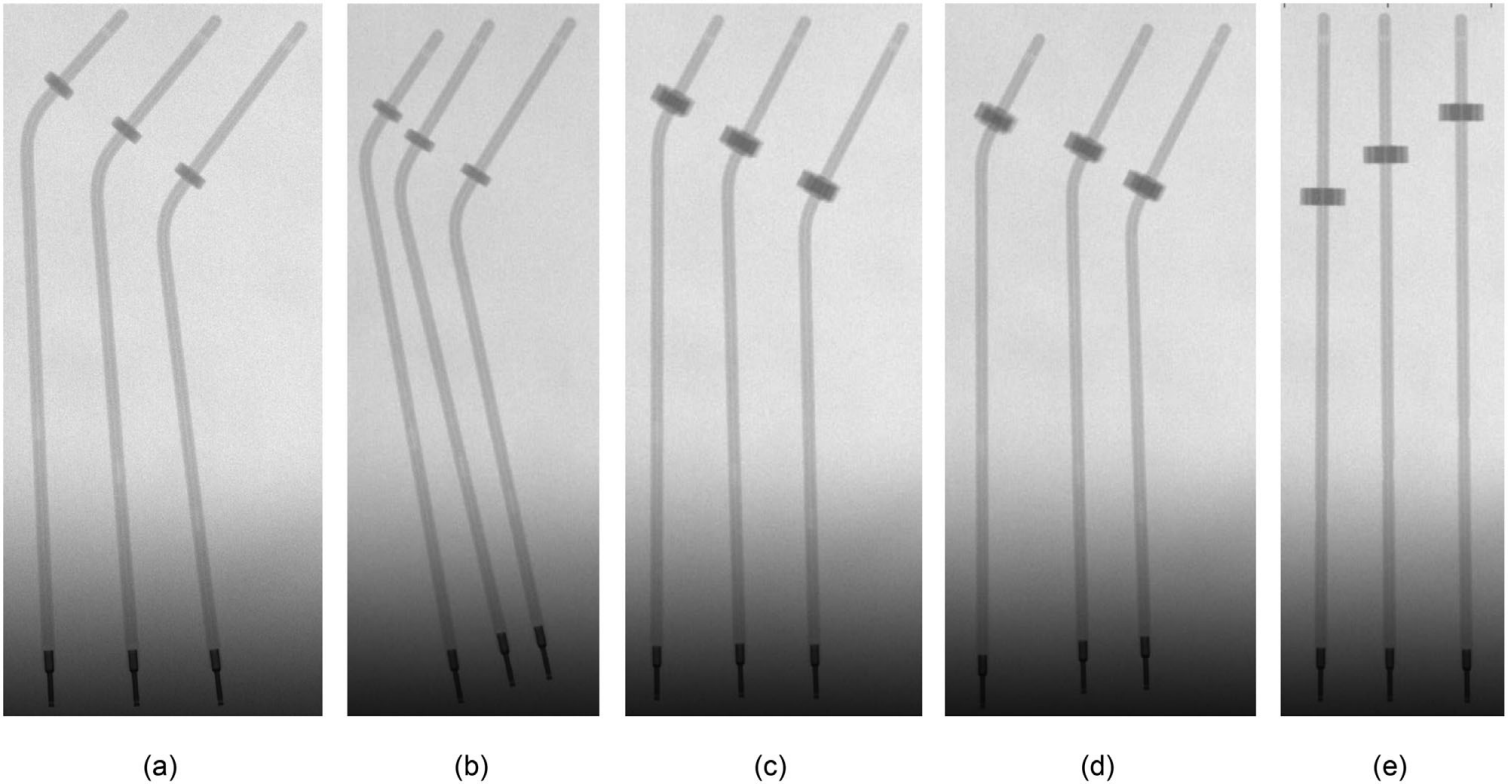
Radiographs of the tandem applicators, with no marker wires in place. Applicators are arranged from left to right, 40 mm, 60 mm, and 80 mm. Shown in (a) are the Set A 45° tandems, (b) the Set B 45° tandems, (c) the Set A 60° tandems, (d) the Set B 60° tandems, and (e) the 90° tandems.

**FIGURE 6:**
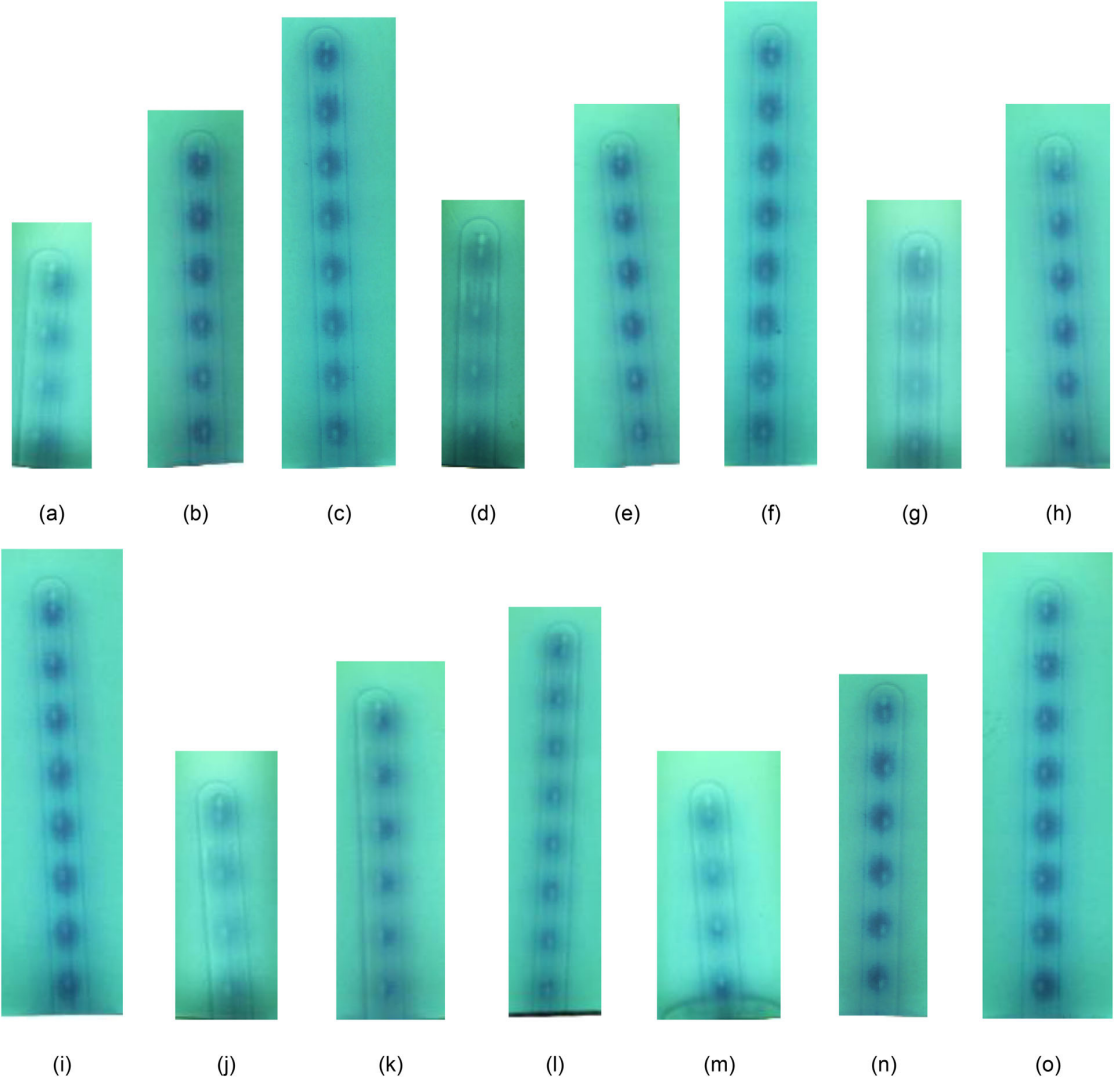
Radiographic and autoradiographic film data of the tandem applicators including the marker wire. Applicators are arranged as follows: (a) Set A 45° 40 mm, (b) Set A 45° 60 mm, (c) Set A 45° 80 mm, (d) Set B 45° 40 mm, (e) Set B 45° 60 mm, (f) Set B 45° 80 mm, (g) Set A 3D 60° 40 mm, (h) Set A 3D 60° 60 mm, (i) Set A 3D 60° 80 mm, (j) Set B 3D 60° 40 mm, (k) Set B 3D 60° 60 mm, (l) Set B 3D 60° 80 mm, (m) 3D 90° 40 mm, (n) 3D 90 mm, (o) 3D 90° 80 mm. The position of the markers on the marker wire from the radiograph was compared to the positions of the source dwells based on the autoradiograph and found to agree to within 1 mm for all dwells in all geometries tested. With the programmed dwells, it is expected that the dark spots should appear overlapping the marker wire positions.

kV radiographic images of the applicators acquired using the on‐board imaging capabilities of a linear accelerator revealed no obvious defects or voids. Images from this process are included in Figures 4 and 5. Autoradiographs, including demarcation using calibrated marker wires, were compared to manufacturer‐provided dimensions for all the tandem probes. The geometry of the tip for all tandem applicators, according to Varian's instructions for use documentation for all sets included in this report, includes a distance of 2.2 mm from the outer tip of the probe to the distal‐most inner lumen point.^15^, ^16^, ^17^ For the uncoded Varian marker wires, the center of the second‐most distal marker indicates the center of the first programmable source position, taking into account the mandatory 1 mm gap between the end of the source channel and the most distal programmable dwell position. Markers in the marker wire are then spaced 1 cm apart for all subsequent positions. Varian indicates that the center of the source is 2.5 mm from the end of the source wire. An average distance from the outer tip of the applicators to the distal‐most inner lumen point was measured to be 2.3 mm ± 0.1 mm, with the reported uncertainty the Type A uncertainty associated with repeated measurements. For these measurements, a distance of 2.2 mm was expected from the tip of the external point of the applicator and the inner lumen as demarcated by the distal‐most marker wire, and then source dwells aligning with subsequent marker positions. The film images confirmed this geometry, as seen in Figure 6.

### 3.3 Offset quantification

**FIGURE 7:**
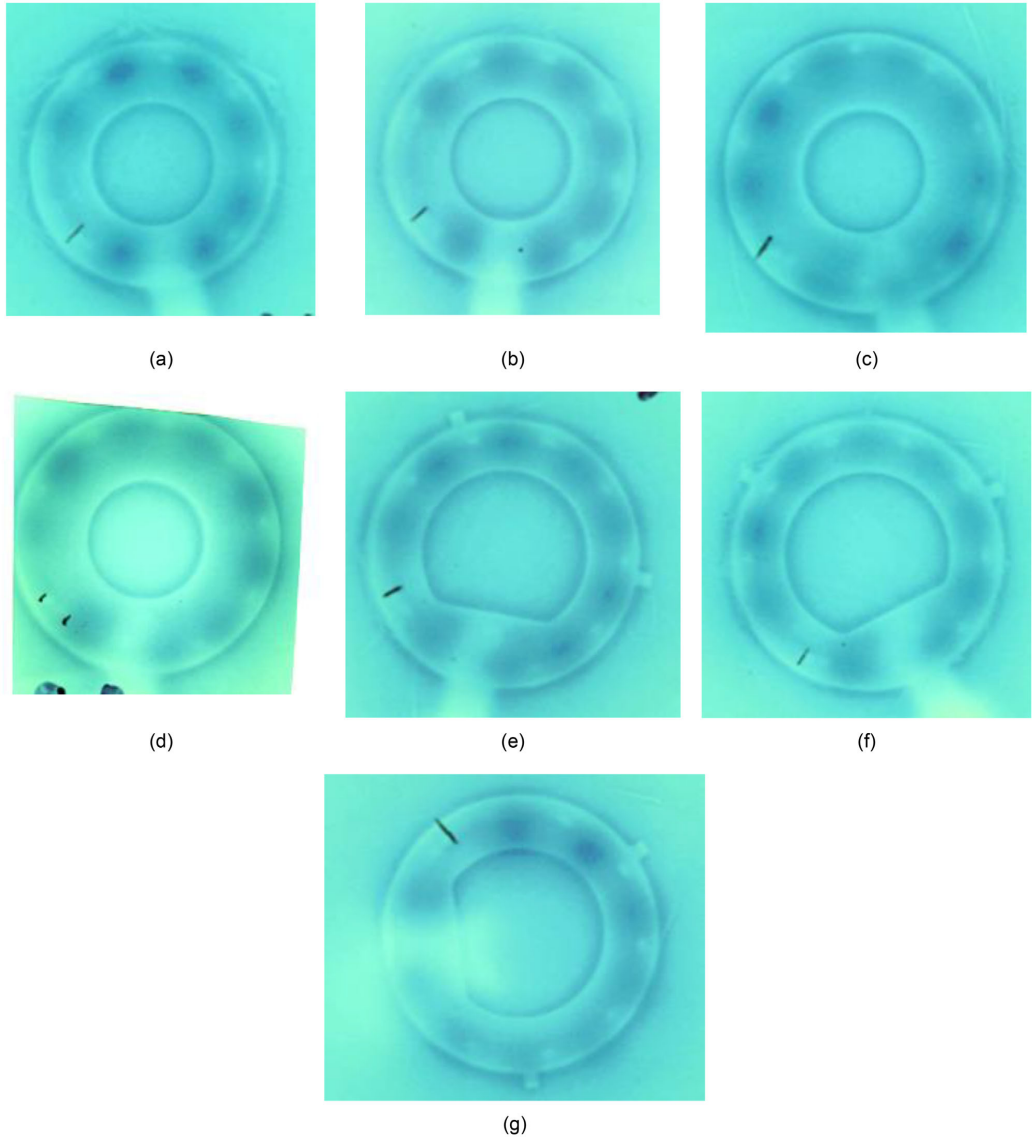
Radiographic and autoradiographic film data of rings using a marker wire with 4 mm offset applied: (a) Set A 45° ring ⌀26 mm × 32 mm, (b) Set B 45° ring ⌀26 mm × 32 mm, (c) Set A 45° ring ⌀30 mm × 36 mm, (d) Set B 45° ring ⌀30 mm × 36 mm, (e) Set A 3D 60° ring ⌀30 mm × 36 mm, (f) Set B 3D 60° ring ⌀30 mm × 36 mm, and (g) 3D 90° ring ⌀30 mm × 36 mm. With the programmed dwells, it is expected that if the applied offset values are correct, then dark spots should appear evenly spaced between marker wire positions.

**FIGURE 8:**
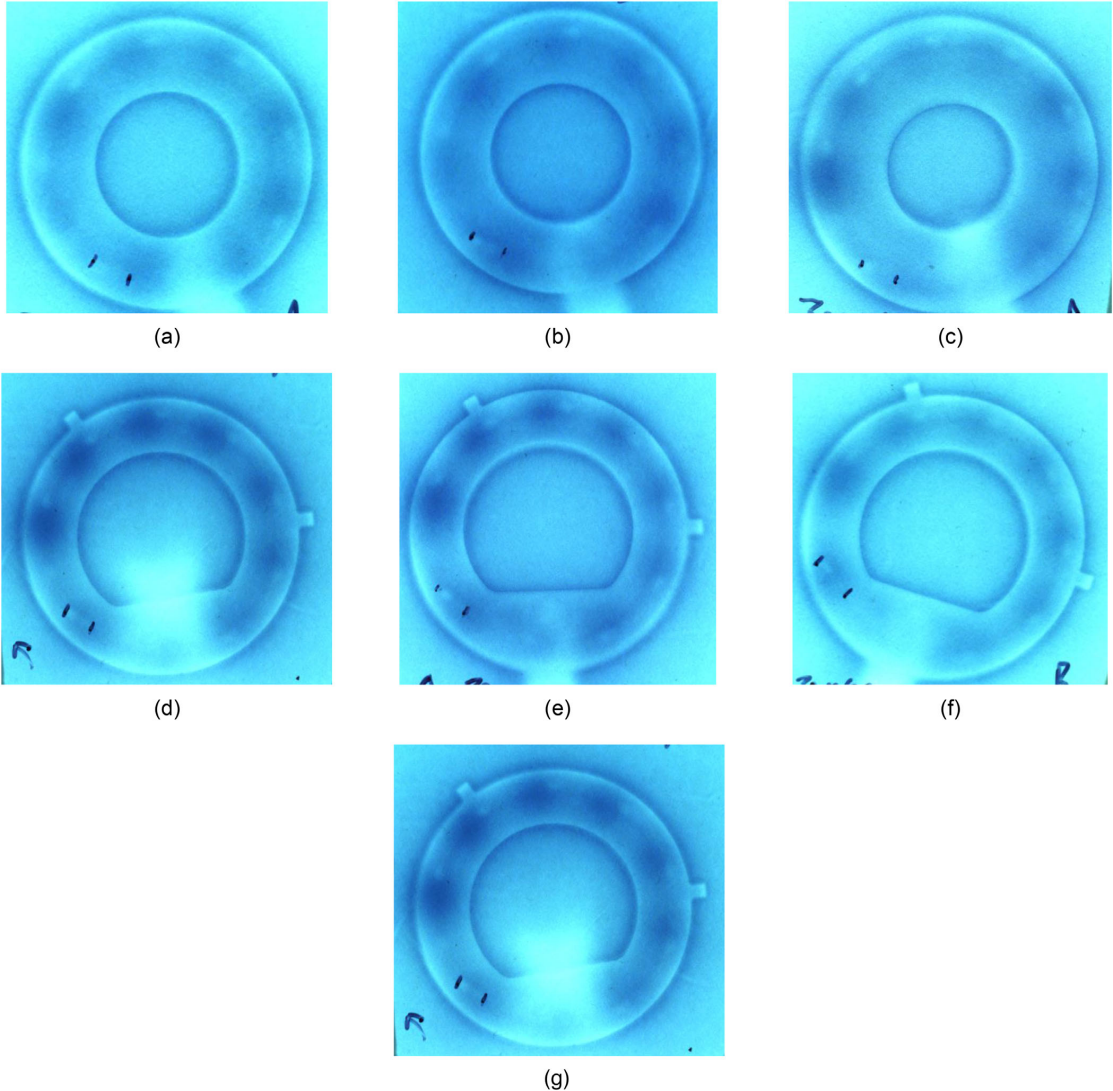
Radiographic and autoradiographic film data of the same rings from Figure 7, acquired using a marker wire with no offset applied: (a) Set A 45° ring ⌀26 mm × 32 mm, (b) Set B 45° ring ⌀26 mm × 32 mm, (c) Set A 45° ring ⌀30 mm × 36 mm, (d) Set B 45° ring ⌀30 mm × 36 mm, (e) Set A 3D 60° ring ⌀30 mm × 36 mm, (f) Set B 3D 60° ring ⌀30 mm × 36 mm, and (g) 3D 90° ring ⌀30 mm × 36 mm.

After repeated measurements, four of the seven rings had initially measured offset values of −0.4 cm, and three of the seven rings had initially measured offset values of −0.5 cm. All rings were verified for a 4 mm shift based on advice from the Varian clinical implementation specialist to use the smallest absolute correction, assuming verification measurements indicated that the lesser magnitude shift rendered clinically acceptable results (Sharon Thompson, M.S., in‐person training communication, September 29, 2020). This verification process was completed by acquiring film radiographs with the marker wire in place, then irradiating the film on the Bravos system for autoradiographic documentation using a 4 mm distal position correction. A 0.4‐second dwell (nominal, for a 10‐Ci source) was programmed at 131.9 cm distal position, then the first significant dwell was positioned at 131.4 cm (a 0.5 cm pull back distance). All subsequent, standard dwell positions within the ring were programmed for 8 seconds, and the first dwell position out of plane was programmed for 15 seconds. With this irradiation geometry, it is expected that if the chosen offset values are acceptable, then dark spots should appear evenly spaced between marker wire positions. It is possible that ring offset values may change over the course of the lifetime of the source wire, with the prospect that after several source deployments, the wire's elasticity may change and conceivably impact ring offset values. Therefore, ring offset values were verified with a used source wire (∼1000 source cycles) as well as a new source wire immediately after a source exchange to compare the offset values over time. Offset values were found not to change based on the age and usage history of the source wire. The initially measured 4 mm offset value was confirmed by these verification measurements. See example film verification data included in Figure 7. Films acquired with no applied offset are included in Figure 8 for comparison.

### 3.4 Ring interrupt testing

It was confirmed that with one or two interrupts, acceptable ring autoradiographs were achieved. As expected, more extensive interrupt testing (ten interrupts in a single run) showed clinically unacceptable results. Based on this result, it was decided at this institution that if multiple interrupts were necessary because of patient movement due to coughing, fatigue, discomfort, etc., or if several interrupts were to be required due to clearance issues, treatment would be discontinued.

### 3.5 CT data and treatment planning system

**FIGURE 9:**
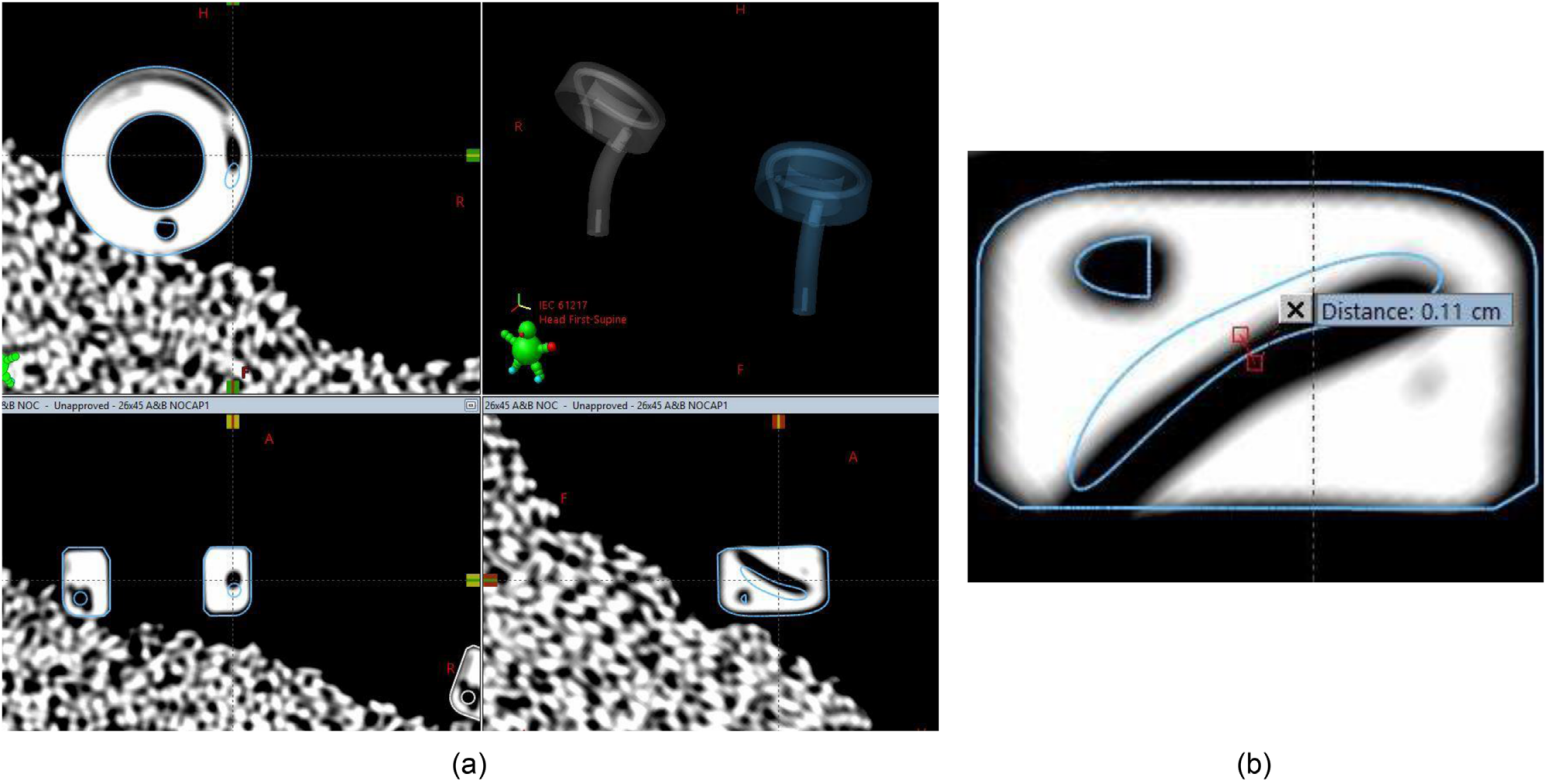
CT images viewed in the treatment planning system of a 45° ring set, ⌀26 mm × 32 mm with no ring cap and no marker wire in place in all three planes in (a) and magnified with distance indicated in (b). The manufacturer‐provided digital 3D model is overlaid with the CT data. The center of the source channel out of the ring plane in the solid model is displaced from the corresponding position in the CT data by approximately 1 mm.

No applicator defects or voids were visible via the CT data. The distance between the outer distal‐most tip of each tandem probe and the inner void indicating the distal‐most point of the inner lumen was measured to be 2.0 mm ± 0.1 mm on the CT data, in agreement with the value provided by the manufacturer as noted in the figure from Varian's instructions for use documentation and the radiographs described earlier. An experienced certified medical dosimetrist and medical physicist reviewed the Varian solid models overlaid on the CT data, and they deemed the models appropriate. Note that for the 45° ring sets ⌀26 mm × 32 mm, the center of the source channel in the solid model deviates from the corresponding position in the CT data by approximately 1 mm in the source channel that is out of plane. This is not expected to have clinical implications based on the expected loading of the rings used at the institution. Example images are included in Figure 9. The source channels in the 45° ring sets ⌀26 mm × 32 mm rings otherwise aligned well with the solid models, and all other applicators aligned very well for the entirety of the solid models. Note that there currently does not exist a “no cap” solid model for the 3D 60° or 90° rings. Varian confirmed this lack of model at the time of commissioning (Sharon Thompson, M.S., in‐person training communication, 29 September 2020).

### 3.6 MRI data

**FIGURE 10:**
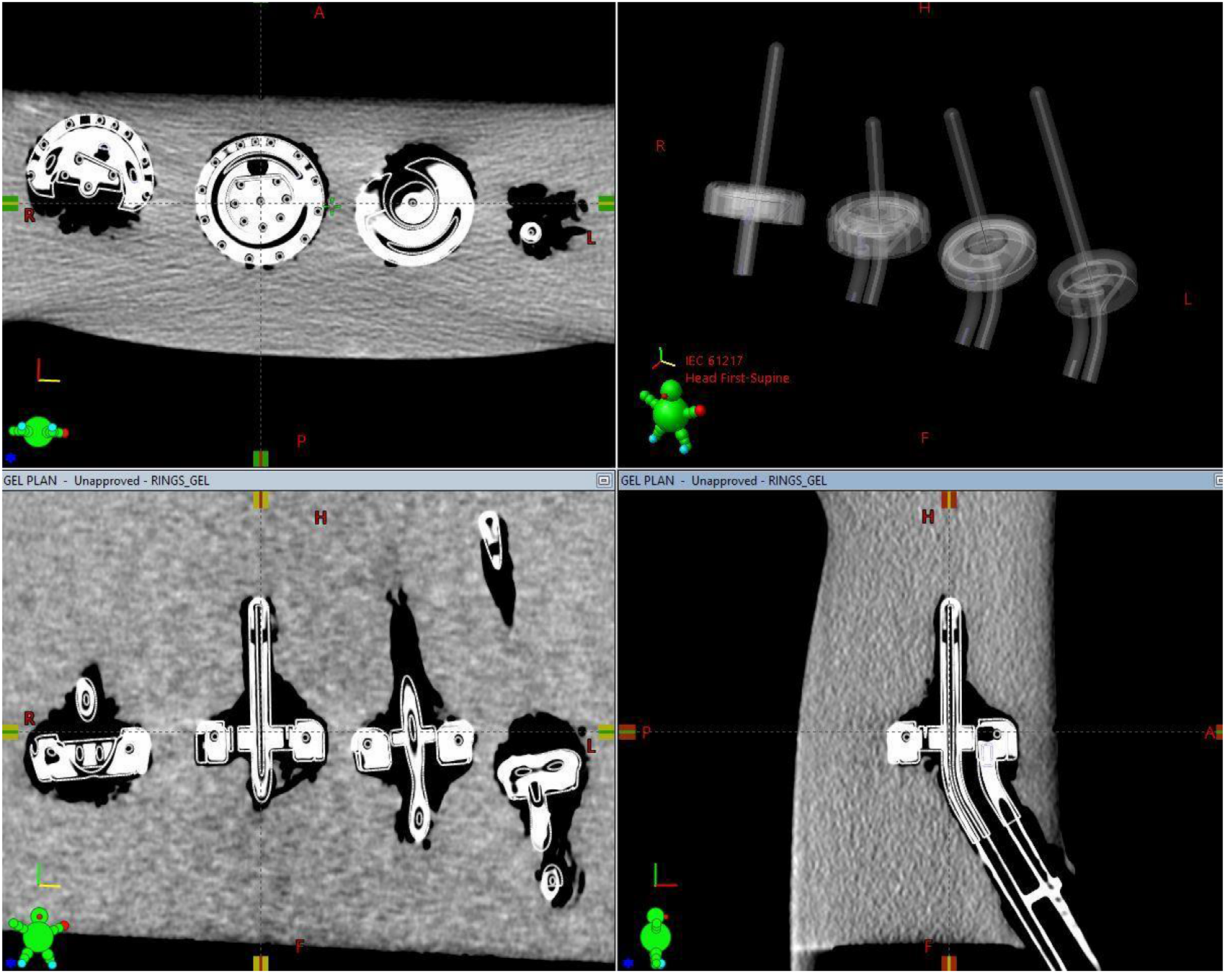
CT images of the gel phantom imported into the treatment planning system, with the manufacturer‐provided digital 3D models of the appropriate applicators overlaid on the CT data. The CT data was rigidly registered to MRI.

**FIGURE 11:**
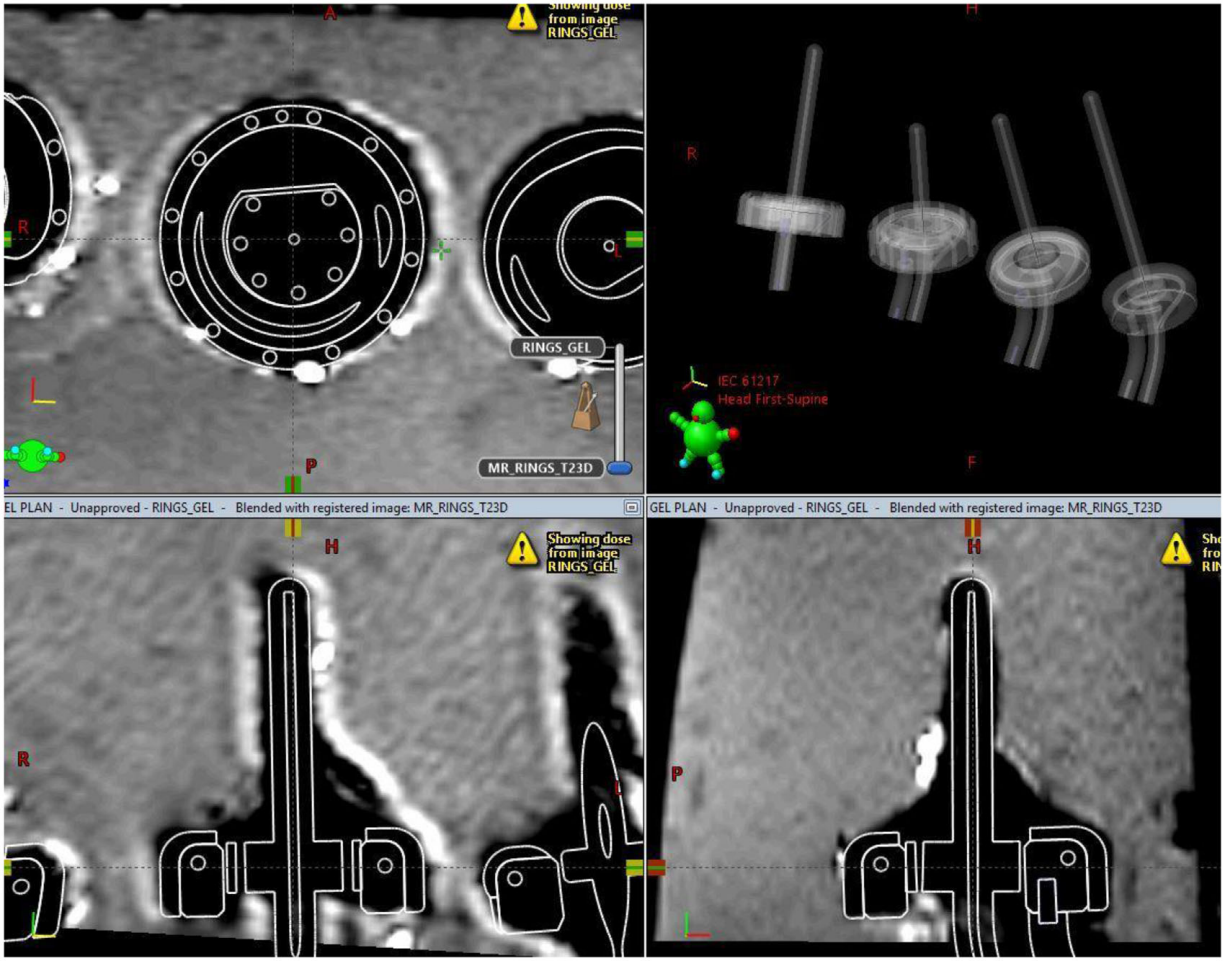
MR images of the gel phantom imported into the treatment planning system, with the manufacturer‐provided digital 3D models of the appropriate applicators overlaid. Shown is the T2 3D sequence.

The MRI scans were rigidly registered with the CT dataset. The image registration was reviewed by an experienced certified medical dosimetrist and authorized medical physicist and deemed appropriate. As recommended by Hellebust et al.,^4^ image registration focused on aligning the geometric position of the applicators between datasets. For this work, minimal distortion was detected and geometric displacement of applicators between rigidly registered CT and MR images was found to be <1 mm for all combinations of MR and CT images. See example images included in Figures 10 and 11. Note that the magnetic susceptibility of the PEEK and titanium applicators was expected to differ from that of the agar gel used in the phantom base, resulting in some expected susceptibility artifact. Air gaps between the agar gel and the plastic‐wrapped applicators were discernible in both CT and MRI images, while susceptibility artifacts were present in MRI only. At this time, the MRI sequences used in this study appear to need some amount of adjustment if MR‐only planning is to be completed, but MR imaging is acceptable for use with CT‐based planning when MRI is used for target delineation only, and not relied on for applicator reconstruction.

## 4 DISCUSSION

This commissioning process for multiple MRI‐compatible ring and tandem applicators reinforced the need for careful assessment of each individual applicator prior to clinical use. At the time of acceptance of the applicators, it was found that one of the 45° ring applicators had “bunched” and unevenly spaced dwell positions in repeated autoradiographic images. This applicator was sent back to the manufacturer following the manufacturer's parts return process, and all data included in this report is for the replacement part. Also found during this process were some slight differences in one ring channel position as denoted in the vendor's 3D applicator model library compared to CT data, and the lack of a “no cap” 3D model for 3D 60° and 90° rings. For these applicators, when measuring distances in BrachyVision from the applicator surface, it is important to ignore the cap included in the solid model if it was not implanted in the patient. This should be clear from the CT data. This underlines the importance of verifying the vendor‐provided library based on its expected clinical use. The forthcoming TG‐236 report is expected to contain recommendations on digital models used in intracavitary brachytherapy treatment planning.

Based on these measurements, it was found that the MRI sequences used for gynecologic brachytherapy cases would require some amount of adjustment if in the future, if it is desired to switch from planning using both CT and MRI acquired with the implant in place, to MRI‐only planning. For the institution's current workflow including MRI used for target delineation and CT used for applicator reconstruction, the tested MRI sequences were found to be adequate. Note that this institution, both a TG‐43 calculation algorithm^18^, ^19^ is available, and Varian's Acuros brachytherapy algorithm, a deterministic grid‐based Boltzmann transport equation solver, is commissioned, following AAPM's TG‐186 guidance.^20^ The TG‐43 algorithm is used for gynecologic brachytherapy planning, including cases involving tandem and rings. Should MRI‐only planning be pursued in the future for cases involving the tandem and ring applicators, only the TG‐43 algorithm would be appropriate without further characterizations.

The ring offset distance characterization is consistent with other similar work. Bellezzo et al.^1^ used an imaging panel to capture Bravos source positioning within one of the rings used in the current work (45° ring, ⌀30 mm × 36 mm). They found a maximum deviation between measured and planned dwell positions of 3.2 mm within the ring, prior to applying any distal dwell correction. Baghwala and Boopathy used titanium, open‐ended ring applicators with Bravos. With the titanium applicators, it is possible to distinguish the outer dimension of the ring (unlike with the PEEK/titanium rings used in this work), but the offset measurement process is the same. They tested 30°, 45°, and 60° rings using film, and for the three rings, found a minimum offset of 3.95 mm, and a maximum offset of 6.42 mm for all dwell positions. They ultimately elected to use a single offset correction of 4.0 mm for all three rings.

**FIGURE 12:**
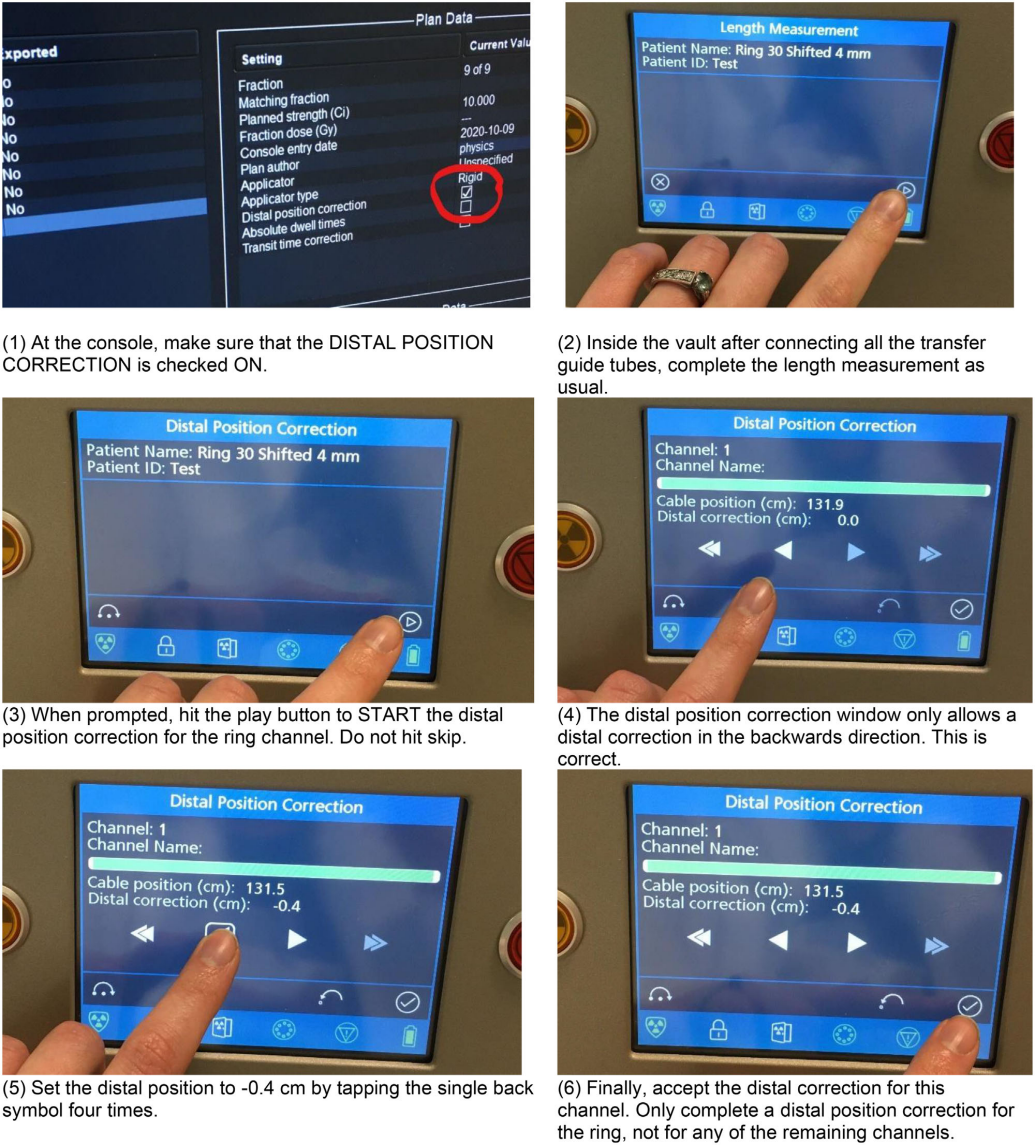
Illustrated process of using the ring sets using the Bravos afterloader touch screen during treatment preparation. For implants including a ring applicator, a distal correction of ‐0.4 cm is required. No other channel uses the distal position correction.

Based on the results of the ring offset distance characterization process described in this work, the following internal usage process was decided. The Bravos system is designed to send the active wire out only as far as the distal‐most programmed position (in contrast to sending the active wire out to the distal‐most possible position), but this commissioning process indicated that the pull‐back process allowed more even spacing, and putting clinically relevant dwell times in the distal‐most possible position resulted in two dwells that were bunched up and unevenly spaced. To assure that the delivered dose distribution matches the planned dose distribution, it was decided to use the Bravos system's distal dwell position correction feature during treatment preparation. When planning with rings, a short dwell is placed at the distal‐most position, which is 131.9 cm for these ring and transfer guide tube combinations (0.4 s for a nominal 10 Ci source). The source is then pulled back 0.5 cm for the first possible clinically usable position of 131.4 cm, and equal spacing of 0.5 cm is used on all subsequent dwells. The plan is planning approved by dosimetry, reviewed by the physician, treatment approved by physics, and sent to the console. Only a single plan is generated, in contrast to the two‐plan process of some institutions. At the console, the physicist verifies that the distal dwell correction is turned on. During treatment preparation, on the afterloader touch screen, the physicist inputs a ‐4 mm position change for the ring channel only. Photographs of this process are included in Figure 12. Training for this process was led by the institution's lead brachytherapy physicist for the entire HDR team, and a hard copy of Figure 12 was posted within the HDR vault and at the HDR console.

Note that the workflow detailed in the previous paragraph and illustrated in Figure 12 is based on commissioning for the applicators included in this report and considering the specific workflows of that institution. As detailed in AAPM's TG‐100 report,^21^ a systematic approach can be used to quantify possible identified failure modes for an institution by evaluating the likelihood of occurrence, the severity of the effect if the failure mode is not caught, and the lack of detectability. Example failure modes and effects analyses (FMEAs) have been published regarding HDR gynecologic brachytherapy (see, for example, Mayadev et al.^22^ and Richardson, Scanderbeg, and Swamidas^23^). These analyses demonstrate the need for design of processes and quality management specific to each clinic. The ring offset process described above illustrates how a set of commissioning measurements may be incorporated into a given institution's practice; however, each user is encouraged to assess individual commissioned applicators in the context of their intended clinical use and their institution's clinical workflows.

## 5 CONCLUSION

Following the processes described here, PEEK and titanium gynecologic HDR applicators, seven rings and fifteen tandems, were commissioned for use with a Bravos HDR brachytherapy remote afterloader. Applicator performance was found to be clinically acceptable considering the clinical environment and workflows for which the applicators are intended to be used, including MRI‐based planning with the MRI acquired with the implant in place. Measurements included channel length, radiographs, autoradiographs, ring offset distances, treatment interrupts, and CT and MRI imaging. A process for applying the measured ring offset correction was established and described. This process leverages the Bravos system's internal distal dwell position correction feature, in contrast to the two‐plan process used at some institutions to account for the ring offset. AAPM's forthcoming TG‐236 report will describe recommended practices when using digital models in intracavitary brachytherapy treatment planning. Prior to the task group report's publication, the vendor‐provided 3D digital models of the tested applicators were reviewed and found to be clinically acceptable in both MRI and CT datasets. It is noted that commissioning was completed in the context of specific planned work processes within this institution's clinical environment. Safety analyses, including failure modes and effects analyses, may help guide decisions regarding required commissioning steps and acceptance criteria for other institutions.

## CONFLICT OF INTEREST STATEMENT

The author declares that there is no conflicts of interest.

## Data Availability

The data that support the findings of this study are available from the author upon reasonable request.
